# Collaborative Mentoring: Perceptions of a sample of Iranian Nursing and Midwifery faculties

**DOI:** 10.30476/jamp.2020.86604.1252

**Published:** 2020-10

**Authors:** HAKIMEH SABEGHI, SEYYED ABOLFAZL VAGHARSEYYEDIN, MARYAM SALMANI MOOD

**Affiliations:** 1 Virtual School of Medical Education and management, Shahid Beheshti University of Medical Sciences, Tehran, Iran; 2 Nursing Department, Birjand University of Medical sciences, Birjand, Iran

**Keywords:** Mentoring, Nursing, Midwifery, Faculty, Qualitative research

## Abstract

**Introduction:**

Mentoring programs have the potential to develop human interaction based on respect, perseverance, and trust. This study aimed to explore the perspectives of a sample of Iranian nursing and midwifery faculty members in Birjand University of Medical Sciences concerning collaborative mentoring.

**Methods:**

Experiences from 17 members were collected using semi-structured interviews. Following the approach recommended by Graneheim and Lundman (2004), a qualitative content analysis was used to analyze the interviews.

**Results:**

Two main themes emerged: ‘reaching team satisfaction through an engaging mentorship’ and ‘group journey toward professional actualization’.

**Conclusions:**

As a feasible intervention, collaborative mentoring sessions can be used to improve personal life, professional interaction, and satisfaction of nursing and midwifery faculty members.

## Introduction

With regard to the critical responsibility of educators in providing quality education and research, it is a critical priority to be up-to-date in order to respond efficiently to the forthcoming academic needs. Beginner educators need not only to be competent in their specialty but also qualified in other interdisciplinary capabilities such as continued education, research, and management as relevant to different areas of medical sciences ( 1 ).

In this regard, novice faculty members are faced with many challenges such as transition to their role as instructor ( 2 ). Moreover, the characteristics of the third millennium students, i.e. change in learning methods and use of new technologies, are among issues that can make the management of classroom highly stressful. In such a situation, nothing is more stressing for the instructor than lack of a mentor to refer to for help and support ( 3 ).

Several propositions have been made to support instructors in transitioning to their roles as instructors in addition to helping them on the way towards scholarship of teaching and learning. The mentoring method has been considered highly efficient in preparing faculty members for their academic and professional roles ( 4 - 8 ). Effective mentoring programs that focus on topics such as career development and modeling can be contributive in moving mentees from an idealist stance to a realistic perspective ( 9 ). Furthermore, mentoring programs in higher education provide an opportunity for the development of human interaction based on respect, perseverance, and trust ( 2 ). 

The concept of mentoring was first introduced in ‘Odyssey’ by Homer. Odysseus entrusted his son, Telemachus, to his friend Mentor to train him the important values and skills and prepare him for adult life ( 10 , 11 ). Mentoring is seen as a human and social relationship, which leads to personal development and entails mutual learning and professional as well as personal growth of the mentor and the learner. This relationship allows experienced practitioners to share their knowledge and experiences with new generations. The mentoring process is regarded as an indicator of the start of the process of self-actualization and growth ( 6 , 9 , 10 ). As defined by the National League of Nursing, mentoring is a key strategy for the establishment of a healthy working environment and promotion and development of faculty members during their professional life as nurse educators ( 12 ). Furthermore, researchers have also shown that mentoring is a major factor in recruitment and retention of the staff ( 10 , 11 ).

Different models have been proposed to be implemented as mentoring programs including traditional or formal models and collaborative (informal) model ( 5 ). In traditional models, the relationship is established between an experienced mentor and one or more mentees. In such a hierarchical relation, the mentor has full authority and power ( 2 ). However, collaborative models, which are often informal, promote teamwork and lead to increased motivation and scientific productivity of the faculty members ( 5 , 9 ). In informal mentoring, the established relationship is unstructured and self-motivated ( 2 , 13 ). 

Formal mentoring is usually characterized with a difference in power hierarchy. Therefore, it is not as easy to establish a relationship between the mentor and the learner. In the collaborative model, the group is shaped voluntarily, and there is a heightened sense of support, cooperation and shared experiences ( 4 ). Therefore, it is obvious that interaction between the members is easily established in the collaborative model. 

Faculty members encounter many stressful situations in the work environment and use different strategies to cope with them. Several previous studies have suggested mentoring to develop the role, professional support, and interrelations between colleagues ( 3 , 4 , 6 ). Positive effects of mentoring have been proposed as empowering faculty members, promoting satisfaction, and providing an opportunity for interaction between novice and senior members ( 2 , 14 ) . 

Given the specific structure of the faculty members of the Nursing and Midwifery Faculty of Birjand University of Medical Sciences (BUMS) (36% novice members and 36% experienced members and 28% mid-careers), collaborative mentoring sessions were held for them. This study aimed to explore the perspectives of a sample of Iranian of nursing and midwifery faculty members in Birjand University of Medical Sciences concerning collaborative mentoring.

## Methods

This study was conducted in Nursing and Midwifery College affiliated to Birjand University of Medical Sciences, Birjand, Iran. The participants were 17 nursing or midwifery faculty members in this college, who had participated in several mentoring sessions. Here, a brief description of these sessions is provided. 

These sessions, which were held as panel discussion and small group sessions, lasted between 45 to 60 minutes on average. For each session a faculty member who had more expertise and experience on the topic was chosen as the leader. The leader gave a speech on fundamentals of the topic, and then all members raised their questions and comments in a quite intimate atmosphere. The newly-employed and experienced members shared their experiences in relation to the challenges posed. The leader made conclusions at the end of each session, and the latest available information based on scientific resources was presented.

The topics discussed in each session are displayed in brief in Table 1.

**Table 1:** Topics of collaborative mentoring sessions

Sessions	Topics discussed
1	Introduction of collaborative mentoring approach and required skills for teamwork
2	Brainstorming used for selection of proper topics and common challenges to faculty members to present in collaborative mentoring sessions
3	Concept of effective teaching and sharing of experiences concerning challenges faced with during their teaching tenure
4	Modern teaching methods and discussion of their application in educational settings given the existing conditions and facilities (2 sessions)
5	Shortcomings and deficiencies of evaluation approaches in clinical settings according to members’ experiences (2 sessions)
6	Introduction of new evaluation methods and approaches to be applied by faculty members (3 sessions)
7	Application of evidence-based practice in theoretical education and clinical settings
8	Manners to hold evidence-based journal clubs by/for students
9	Characteristics of the students of the third millennium
10	Challenges of millennial students and strategies to guide them and manage classes
11	Introducing appropriate approaches for the teaching-learning process such as Harden Educational Strategy
12	Discussion about research ideas in education

To explore the perspectives of nursing and midwifery faculty members concerning collaborative mentoring, a qualitative approach, using qualitative conventional content analysis, was applied. Data collection and analysis was done from September 2017 to September 2018. Purposive sampling was used to select the participants. Maximum variation sampling strategy was applied with the members’ gender, age, tenure, and fields of specialty. The subjects then participated in semi-structured interviews, using the following questions: “Will you elaborate on your experience of participating in the meetings at the faculty?”, “Will you talk about the effects of participating in the meetings in terms of your professional and personal life?" Based on your personal experience, “Will you comment on your preference for continuation or termination of such sessions?”, “How did you feel about participating in this sessions?”, and “What changes have these sessions made in your academic life?”

Then, according to the responses provided, some probing questions were asked. The probing questions were not a fixed series of questions but they were based on the unique responses by the participants.

The semi-structured interviews lasted from one to two hours. Within a maximum of 24 hours, the interviews were transcribed verbatim and read in order to come to a general idea of the experiences of the interviewees. Totally, 17 members of the faculty were interviewed. Since the last three interviews provided no new categories, the team felt the data were saturated. Since it is suggested that reaching data saturation can indicate an appropriate sample size ( 15 ), the process of data collection was stopped at this stage, and the data were analyzed.

Building on the approach recommended by Graneheim and Lundman (2004) ( 16 ), the interviews were first coded line by line. The codes were compared based on their similarities and differences and arranged in the form of sub-categories, which were later grouped according to similar meanings. Finally, given the original concept of categories, two themes emerged from the data analysis. MAXQDA software was used to facilitate data management.

### Rigor

The interviews were analyzed by two researchers independently for conformability. Afterwards, the results were compared, while modifications were made in order to reach consensus. Peer checking and member checking were also used ( 17 ). 

One of the ways to ensure credibility is long-term involvement with the subject under study. The researchers were present in the sessions as faculty members before the start of the study; they had a strong interaction and relation with the participants both before and after the study.

In this study, the researchers tried to increase research credibility with long and deep involvement with data as well as checking the findings by the participants. Similarly, trustworthiness was enhanced using different data collection methods (interviews, observations and memos). In addition, it was tried to accurately record and report the steps and decisions taken to facilitate follow-up researches by others.

### Ethical consideration

The proposal of the present research was approved by Research and Ethics Committee of the Birjand University of Medical Sciences (Ethical ID: IR.BUMS.REC.1395.302). Before conducting the interviews, the potential participants were provided with written information on the objectives of the study. They were informed that their participation in the study was voluntary and they could leave the study at any time without penalty. Written informed consent was obtained from them regarding their participation in the study. Also, they were assured that the collected data will remain strictly confidential and will not be available to anyone other than the research group.

## Results

The Participants (6 men and 11 women) had a mean age of 37.5 years (ranging from 28 to 52 years) and were either assistant professor (n=5) or instructor (n=12) with an average work experience of 7.8 years (ranging from 1 to 28 years). 

Analysis of the interviews led to the emergence of two main themes: ‘Reaching team satisfaction through an engaging mentorship’ and ‘Group journey toward professional actualization’. The first theme covered three main categories including "Promotion of life quality", "Encouragement of members to active participation", and "Focus on goal-oriented design of sessions". The second theme consisted of three categories: "Promotion of professional competence", "Facilitating interactions between faculty members", and "Overall satisfaction and emphasis on continuing the meetings". The relevant themes, categories and their subcategories along with sample quotes of the participants are presented in Table 2.

**Table 2:** Themes, Categories, Subcategories and Quotes of Participants

Sub-categories	Categories	Themes
Getting away from daily life	Promotion of life quality	Reaching team satisfaction through an engaging mentorship
The impact on members’ psychosocial aspects of life		
Enhancing the morale of members		
Creating a platform for active participation of the members at the sessions	Encouragement of members to active participation	
Establishment of logical balance between formality and informality of the sessions		
The need for diverse, attractive and applicable topics		
The importance of considering the interests and preferences of individuals in organizing sessions		
The need for systematic design of meeting	Focus on goal-oriented design of sessions	
The necessity to criticize the discussed topics to enrich the educational-research dimension of the meetings		
The need for the attractiveness of the way sessions are held		
Allowing participation of members of other departments to assist in enriching the sessions		
Promotion of educational competence of the individuals	Promotion of professional competence	Group journey toward professional actualization
Inventing new research ideas in the sessions		
Helping the scientific promotion of colleagues’ academic rank		
Increased likelihood for solving the challenges of professional members after the creation of an intimate atmosphere and effacement of the hierarchy of power	Facilitating interactions between faculty members	
The impact of the informal nature of the meetings on the free expression of professional experiences		
Facilitating sharing of experiences between colleagues		
The role of meetings in the transfer of professional experiences of experienced faculty to novice members and vice versa		
Role of meetings in the development of professional interaction and improvement of interpersonal communication		
Facilitating the teaching-learning process due to the amiable atmosphere of the meetings	overall satisfaction and emphasis on continuing the meetings	
Satisfaction with attendance in the sessions		
Emphasis on productivity of the sessions		

Regarding the first theme, i.e., ‘reaching team satisfaction through an engaging mentorship’ faculty members stated that these meetings paved the way to enhanced cooperation between them. Generally, in the theme ‘group journey toward professional actualization’, the participants believed that their participation in the peer mentoring sessions facilitated the process of interaction between them. The participants both expressed their satisfaction with the sessions and their stress on continuation of the sessions, and generally acknowledged the promotion brought to their professional capabilities.

## Discussion

This study aimed to explain the experiences of nursing and midwifery faculty members about collaborative mentoring programs.

### Reaching team satisfaction through an engaging mentorship

The first theme was “Reaching team satisfaction through an engaging mentorship," which included "Promotion of the quality of life", "Encouragement of members to active participation", and "Focus on goal-oriented design of sessions".

As the participants pointed out, the sessions improved the relationships between colleagues. One of the participants stated: “... This meeting was an opportunity for the colleagues to work together without any concerns and preoccupations”. Research has proposed that social relationships have a positive impact on mental health, health behavior, as well as physical health ( 18 ). Consequently, it is expected that the participants' quality of life is positively improved by participating in mentoring sessions. The results of a study on the medical school faculties in the University of Massachusetts also indicated that the participants regarded the collaborative mentoring program as a valuable means for their personal growth ( 19 ).

Faculty members also believed that these meetings provided a platform for an active cooperation between them. In fact, active participation, mutual support and collaborative learning are three essential components of success in collaborative mentoring program ( 20 , 21 ). If managers are to enhance partnerships between faculty members, successful application of collaborative mentoring can be an effective tool.

Referring to some useful tips to improve sessions, many faculty members investigated here asserted that for further fruitfulness of meetings, it is essential that planned, systematic and goal-oriented sessions be held. In line with this finding of the study, after determining the original values of the members in collaborative mentoring approach, short and long-term career goals are based on the identified values. 

### Group journey toward professional actualization

The theme ‘Group journey toward professional actualization’ had three categories:" Promotion of professional competence", "Facilitating interactions between faculty members", and "Overall satisfaction and emphasis on continuing the meetings.” The participants believed that holding the meetings, in addition to facilitating the process of interaction between them, leads to improved professional capabilities. 

The results of this study also revealed that due to intimate atmosphere of sessions and dissolution of the hierarchy of power, the probability for solving the members’ professional challenges was increased. Compatible with this finding of the current research, Pololi, et al. (2002) also refer to the non-hierarchical nature of peer-mentoring process as an advantage compared with features such as power, dominance, dependency and transference in the official mentoring methods ( 19 ).

The participants of this study posited that the informal nature of the meetings led to the free expression of professional experiences and facilitation of sharing common experiences between their colleagues. In other words, such a feature of the meetings results in the development of professional interactions and improved interpersonal communication.

Studies have also shown that participants in the mentoring programs feel more comfortable sharing information with each other and face fewer restrictions when discussing a topic of a professional nature. In fact, for the inherent equality between the members of the group, the relationships are mutual and there is usually a valuable thing for every person to share or learn ( 22 ). Therefore, since establishing a relationship in informal mentoring method is easier and more meaningful between the team members compared to communication in an official method ( 19 ), it increases the possibility to express opinions and share experiences that in turn will play an important role in facilitating learning and will eventually cause satisfaction of the participants with the meetings. Such a finding has been confirmed in other studies conducted on collaborative mentoring as well ( 22 ).

Based on the available evidence, mentoring can best be applied in an environment with positive relationships where people can trust each other. In such environments individuals are enabled to express their opinions freely and their learning is facilitated ( 5 ). 

In this study, mentoring sessions enhanced the professional capabilities of faculty members in areas such as education and research activities. Previous studies have shown that new developments in health care delivery methods, faculty members’ lack of time and limited support of universities for scholarly activities and teaching, and lack of good mentors can reduce faculty members’ scholarly productivity and job satisfaction ( 5 ). It seems that a highly effective approach to respond to these challenges can be collaborative mentoring ( 22 ). In addition to novice instructors, mentoring are also beneficial to mid-career and experienced faculty members. For mid-career faculty members, mentoring programs can be a perfect opportunity to update their information and ideas about teaching. For experienced faculty members, these programs are opportunities to mentor others, get socialized and return back to the academic community ( 23 , 24 ). 

According to Pololi and knight (2005), the best mentoring practices appear in an environment with positive relationships within an atmosphere where there is trust between people, and this facilitates learning and people can freely express their opinions ( 19 ). Such a setting will motivate people to collaborate in determining the values, priorities and their learning needs ( 5 ). The results of a study in 2009, conducted at 17 nursing schools, demonstrated that using collaborative mentoring program increased the academic productivity of instructors ( 25 ).

One of the most important factors affecting the learning process is the learning environment. Collaborative mentoring, as one of its merits creates a safe and supportive learning environment that facilitates communication. The results of building a safe learning environment that enhances interaction between the participants include improving the relationships between colleagues, sharing experiences, partnership and team collaboration and mutual problem solving ( 19 ).

Given the qualitative nature of this study, the results of this research cannot be generalized to similar populations in other schools of nursing and midwifery in different regions of Iran. Moreover, only a single nursing and midwifery college was selected for this research. Thus, the findings of present may not be applicable to other nursing and midwifery colleges.

## Conclusion

The results of the present study revealed the effectiveness of collaborative mentoring sessions on personal life, professional interaction and satisfaction of the participants of the study.

These findings can also serve as a ground with which results of other studies about the collaborative mentoring in different parts of the world can be compared. A comparison of the results of the collaborative mentoring at different time intervals is recommended to be conducted in the future studies.

## References

[1] Dansfahani Z, Javadi M, Zohal M, Ziaee A, Yazdi Z, Zahedifar F, et al ( 2013). Training needs assessment of empowerment of faculty members in Qazvin University of Medical Sciences based on regulations of empowerment and increasing the knowledge of faculty members plan. The journal of medical education & development.

[2] Savage HE, Karp RS, Logue R ( 2004). Faculty Mentorship at Colleges and Universities. College Teaching.

[3] Dhed Msn AM, Michelle M ( 2013). Mentoring new faculty. Procedia-social and behavioral sciences.

[4] Anibas M, Brenner GH, Zorn CR ( 2009). Experiences described by novice teaching academic staff in baccalaureate nursing education: a focus on mentoring. Journal of professional nursing: Official Journal of the American Association of Colleges of Nursing.

[5] Heinrich KT, Oberleitner MG ( 2012). How a faculty group's peer mentoring of each other's scholarship can enhance retention and recruitment. Journal of professional nursing: Official Journal of the American Association of Colleges of Nursing.

[6] Specht JA ( 2013). Mentoring relationships and the levels of role conflict and role ambiguity experienced by novice nursing faculty. Journal of professional nursing: Official Journal of the American Association of Colleges of Nursing.

[7] Anderson JK ( 2009). The work-role transition of expert clinician to novice academic educator. The Journal of nursing education.

[8] Clark NJ, Alcala-Van Houten L, Perea-Ryan M ( 2010). Transitioning from clinical practice to academia: university expectations on the tenure track. Nurse educator.

[9] Mullen CA, Klimaitis CC Defining mentoring: a literature review of issues, types, and applications, Annals of the New York Academy of Sciences. New York: Academy of Sciences; 2019.

[10] Kowtko C, Watts LK ( 2008). Mentoring in health sciences education: A review of the literature. JMIRS.

[11] Sowan NA, Moffatt SG, Canales MK ( 2004). Creating a mentoring partnership model: a university-department of health experience. Family & community health.

[12] Hubbard C, Halcomb K, Foley B, Roberts B ( 2010). Mentoring: A nurse educator survey. Teaching and Learning in Nursing.

[13] Tourigny L, Pulich M ( 2005). A critical examination of formal and informal mentoring among nurses. The health care manager.

[14] Roets L, van Rensburg EJ, Lubbe J ( 2019). Faculty’s experience of a formal mentoring programme: the perfect fit. African health sciences.

[15] Guthrie J, Petty R, Yongvanich K, Ricceri F ( 2004). Using content analysis as a research method to inquire into intellectual capital reporting. Journal of Intellectual Capital.

[16] Graneheim UH, Lundman B ( 2004). Qualitative content analysis in nursing research: concepts, procedures and measures to achieve trustworthiness. Nurse education today.

[17] Polit DF, Beck CT Essentials of Nursing Research appraising evidence for nursing practice. 8th ed. New York: Lippincott Williams & Wilkins; 2014.

[18] Umberson D, Montez JK ( 2010). Social relationships and health: A flashpoint for health policy. Journal of health and social behavior.

[19] Pololi LH, Knight SM, Dennis K, Frankel RM ( 2002). Helping medical school faculty realize their dreams: an innovative, collaborative mentoring program. Acad Med: Journal of the Association of American Medical Colleges.

[20] Kairat M ( 2019). Informal faculty mentoring practices in higher education in Kazakhstan. Journal of Education in Black Sea Region.

[21] Sprengel AD, Job L ( 2004). Reducing student anxiety by using clinical peer mentoring with beginning nursing students. Nurse educator.

[22] Bussey-Jones J, Bernstein L, Higgins S, Malebranche D, Paranjape A, Genao I, et al ( 2006). Repaving the road to academic success: the IMeRGE approach to peer mentoring. Acad Med: Journal of the Association of American Medical Colleges.

[23] Hunt CW, Ellison KJ ( 2010). Enhancing faculty resources through peer mentoring. Nurse educator.

[24] Huston Th, CL W ( 2008). Peer coaching: professional development for experienced faculty. Innov High Educ.

[25] Maas ML, Conn V, Buckwalter KC, Herr K, Tripp-Reimer T ( 2009). Increasing Nursing Faculty Research: The Iowa Gerontological Nursing Research and Regional Research Consortium Strategies. Journal of nursing scholarship: an official publication of Sigma Theta Tau International Honor Society of Nursing / Sigma Theta Tau.

